# Depressive Symptoms Effect on Self Care Behavior During the First Month After Myocardial Infarction

**DOI:** 10.5539/gjhs.v7n4p382

**Published:** 2014-01-25

**Authors:** Maryam Niakan, Ezzat Paryad, Ehsan Kazemnezhad Leili, Farzane Sheikholeslami

**Affiliations:** 1 Shahid faghiihi Educational and Therapeutic Center, Shiraz University of Medical Sciences, Shiraz, Iran; 2 Social Determinants of Health Research Center (SDHRC), School of Nursing and Midwifery, Guilan University of Medical Sciences, Rasht, Iran; 3 School of Nursing and Midwifery, Guilan University of Medical Sciences, Rasht, Iran

**Keywords:** depression symptoms, myocardial infarction, self care behavior

## Abstract

**Aim:**

To determine the effect of severity of depression symptoms on self care behavior in 15th and 30th day after myocardial infarction (MI).

**Materials and Methods:**

Gathering data for this cross sectional study was done by Beck depression and self care behavior questionnaires in a heart especial hospital in Rasht in north of Iran. Sample size was 132 after MI patients and data collected from June 2011 to January 2012.

**Results:**

Scores of depression symptoms in 15th and 30th day after MI and score of self care behavior in these days had significant difference (P<0.0001). Spearman test showed self care behavior had significant relationship with depression symptoms (P<0.0001). GEE model also showed with control of socio demographic and illness related factors, depression symptoms can decrease self care behavior scores (P<0.001).

**Conclusion:**

Severity of depression symptoms increase in 15th to 30th day after MI. This issue can affect on self care behavior. This issue is emphasized on nurses’ notice to plan suitable self care program for these patients.

## 1. Introduction

Self care behavior in chronic diseases patients is a one of the effective factors on their health and symptoms. Coronary artery disease (CAD) is a chronic disease and self care behavior can help to reduced severity of its signs and symptoms (Sidani, 2003).

The most severe stage of CAD is myocardial infarction (MI) and it influences on self care behaviors, that assist individuals in minimizing the progression of CAD (Coyle, 2009). Self care behavior after MI include adherence to drug regimen, control of diet, exercise performance, reduction of stress and cessation of smoking (Nikpajooh, 2009) finding of a study in Japon showed the main factor in readmission of chronic patients is not to do self care behaviors (Kato et al., 2009). Many of factors can influence adherence and self care behaviors (Cameron et al., 2009). Patient knowledge about effects of self care behaviors on her or his outcomes (Riegel & Carlson, 2002), previous education about self care (Van der wal et al., 2006) and depression symptoms (Van der wal et al., 2006; Parashar & Vaccarino, 2007) are factors can related to self care behaviors.

Depression is a powerful factor which affect on self care behavior (Wulsin & Singel, 2003; Linke et al., 2009; Linke et al., 2005). Frequency of depression symptoms after MI in Iran is 52 % (Modabernia et al., 2001), meanwhile finding of a study about symptoms after MI showed, only 42.2 % of patients after MI experienced depression (Ellis et al., 2005). Despite of importance of exact depression symptoms time occurrence after MI, findings of different studies don’t have similarity in time of depression symptoms beginning after MI. In the most of studies the time of this symptom beginning was reported in 3, 6, 8 and 24 months after MI (Hosseini et al., 2005; Dickens et al., 2007; Spijkerman et al., 2006; Kronish et al., 2006; King, 2001; Strik et al., 2001). Furthermore a few of studies reported the severity of depression symptoms during the first month after MI (Hosseini et al., 2005; Bender, 2009; Lauzon et al., 2003), meanwhile finding of Morgan et al. (2009) showed frequency of depression was 34 % in 3-6 month after MI. It is noticeable the most time of the reinfarction or death due to infarction is within the first six months after MI (Lane et al., 2001; Mayou et al., 2000) and patients who do not adhere to prescribed regimens will more likely to die within a year after MI (Coyle, 2009). Occurrence of depressive symptoms after MI effect on patients behaviors (Parashar & Vaccarino, 2007). Patients with depression symptoms don’t like to participate in rehabilitation programs and their attendance to adhere to nutrient and drug regimens and cessation of smoking are decreased (Coyle, 2009; Sheikholeslami, 2002).

In summary many of previous studies have found different findings about the beginning time of depression symptoms after MI and Iranian researchers don’t have evaluate the common these symptoms beginning time. The findings of this study can be used to increase notice to rehabilitation programs to preparing suitable situations for self caring in patients after MI.

*The Study Aim*

The purpose of this research was:

1. To determine depressive symptoms severity during the first month after MI;
2. To determine self care behavior during 15th and 30th day after MI;
3. To evaluate relationship between depression symptoms and self care behavior;
4. To predict severity of depression symptoms effect on self care behavior with demographic and illness related factors control.

## 2. Materials and Methods

This study has a cross sectional approach and the data was collected from June 2011 to January 2012.

### 2.1 Location

The environment of this study was cardiac wards and special clinic of heart diseases in a special hospital in Rasht in north of Iran.

In this center, patients with MI diagnosis are admitted in CCU setting. They transferre to cardiac wards 5-7 day after infarction. The reason of choosing these wards, was avoiding speaking with MI patients in critical situation in CCU, so these wards were suitable for collecting data. All of the patients after discharging of hospital has to come back to special clinic for physician evaluation. Thus this location was suitable for collecting data.

### 2.2 Participants

A convenience sample of 132 patients with MI diagnosis who transferred from CCU to cardiac ward was studied. Detection of sample size was done based on correlation between depression symptoms and self carebehavior in Coyle (2009) study.

The inclusion criterions consisted of age more than 18 and ability to writing and reading in Persian. All of the MI patients who have MI diagnosis were recorded in their medical document and not to have history of psychiatric drug use based on their medical ducuments and themselves expression, eligible for participating in this study.

In the second step in 15th day after MI 18 eligible MI patients didn’t come back to heart clinic, thus other 18 eligible patients entered to study as sample. All of these patients (132) came back to clinic in 30th day after MI

### 2.3 Questionnaires

1). Socio-demographic and illness related factors questionnaire prepared based on review of literatures and previous similar studies. This section had items about age, sex, married status, job, health assurance status, income, living place, psychiatric drug history, ADL doing ability before MI, hypertension and smoking history.
2). Beck depression inventory II, that measure depression symptoms (Beck et al., 1996). This questionnaire has 21 items. Maximum of its score is 63 and achievement 0-9 scores is showed minimum symptoms of depression and achievement 30 – 63 scores is showed severe depression symptoms.
3). Self care behavior questionnaire. For preparation of this section, Miller (1982) self care behavior questionnaire was used. This tool was used after that by Coyle. This questionnaire has 20 items about drug regimen, control of diet, exercise performance, stress modifying and smoking cessation rated on a 5- pointed Likert scale ranging from 20 to 100, which the highest score indicates the better self care behavior. Each domain has 4 questions about self care.
4). Charlson co morbidity questionnaire was used to evaluate effect of co morbidity on self care behavior. This tool made by Charlson in 1987 and evaluating effect of 19 chronic illnesses by score detecting. Scores from this tools adjust by age by Excel software, which the highest score indicate highest effect of co morbidity on self care behavior.

### 2.4 Validity and Reliability

The psychometric evaluation of Back depression questionnaire was done in Iran by Rajabi et al., (2001), so in this research, we do not detect validity and reliability of this tool, but about self care behavior scale, at first this questionnaire translated to Persian and retranslate to English by an English language expert, after that the Persian version sent to an expert panel, consisted of 16 nursing experts, they filled CVR and CVI form for this questionnaire and all of its items achieved more than 80% of score. For internal consistency detection of this questionnaire we detected Alpha Chronbach. In these step 20 questionnaires was filled by patients after MI in a pilot study. Alpha value for control of diet, smoking cessation, exercise performance, drug regimen and stress modifying were in order 0.90, 0.98, 0.81, 0.92 and 0.80.

### 2.5 Ethical Consideration

This study was approved by Guilin University research ethics committee in north of Iran. Eligible patients were given written and oral information about the study and asked them to give written consent to participate confidentiality was assured. In consent from was written participation is voluntary and they can go out of study in each step.

### 2.6 Data Collection

The questionnaire was used for data collection in three steps. In first step the eligible patients in cardiac ward trained by one of the research team about self care behavioral domains what would be asked in questionnaire. A booklet and educational film was used for each of eligible behaviors. Thus all of the samples had similar training about self care after discharge. In this step demographic and Charson questionnaires were filled and the other data consisted of the Beck depression questionnaires and self care behavior questionnaires were filled in 15th and 30th day after MI. For referring patients in exact time, 2-3 day before refer date, all samples received a phone call.

### 2.7 Data Analysis

Analyzing of collected data was done by SPSS version 19. After coding and entering data, in first step Kolmograph Smirnove test showed data did not have normal distribution so Mann withney U and kruskalwalis tests were used for detecting correlation of depression symptoms and self care behavior with demographic and illness related factors. For comparing depression symptoms in 15th and 30th day after MI, Wilcoxon test was used and Spearman test was used to detect correlation between depression symptoms and self care behavior. After that for detecting effect chance of depression symptoms on self care behavior generalize Estimated Equation (GEE) was used. This model was applied in studies which dependent variable in different situation, accept different amount. In this study GEE model determined the dependency severity of self care behavior to depression symptoms in 15th and 30th day after MI. In addition this model was used to predict the effect chance of depression symptoms on self care behavior with control of demographic and related illness factors. All statistical tests significance of P value was < 0.05.

## 3. Results

In the first step 150 admitted patients after MI filled consent form in hospital and participated in educational program after that, 18 patients didn’t refer to clinic in 15th day after MI, thus 132 patients participated in three steps. Their demographic data is shown in Table 1.

**Table 1:** Demographic characteristics of participants (n=132)

Variables	Number	%
Age		
<65	92	69.7
>65	40	30.3
Mean	59.92+/-10.68	
Gender		
female	64	48.5
male	68	51.5
Marital status		
single	1	0.8
married	106	83.3
divorced	2	1.6
missing	23	17.4
Education		
<high school diploma	100	75.8
≤high school diploma	32	24.2
Health assurance		
yes	121	91.7
no	11	8.3
Residence status		
urban	71	53.8
rural	61	46.2
Job status 2 weeks after MI		
yes	57	43.2
no	75	56.8
Job status 4 weeks after MI		
yes	88	66.7
no	44	33.3
MI history times		
No MI history	88	59.8
One time	22	23.6
Two times	12	9.1
Three times	1	0.8
Four times and upper	9	6.8
Hypertension histoty		
yes	76	57.6
no	56	42.4
Smoking history		
Yes	56	42.4
no	76	57.6
Charson comorbidity score		
1-2	42	31.8
3-4	66	50
≥5	24	18.2

Depression symptoms after discharge

In the 15th day after MI, the majority of samples had mild depression symptoms (43.2%) but in 30th day the majority of participants (51.5%) had moderate symptoms of depression. It is noticeable, in 15th day 37.9 % of participants didn’t have depression symptoms but in 30th day all of them had these symptoms. Wilcoxon test showed difference between depression symptoms in 15th and 30th day after MI was significant (P< 0.0001). This test also showed significant relationship between depression symptoms and all of the demographic variables. Mannwitney U test showed severe depression symptoms based on demographic variables such as gender, assurances, living in city or village, history of previous MI, hypertension and smoking history have significant difference, and meanwhile this deference was not significant in 30th day after MI. In this day only associated chronic diseases had significant effect on severity of depression symptoms (P < 0.04).

72 % of these study participants had desire self care behavior in 15th day after MI, meanwhile in 30th day only 15.9 % of them had desire self care behavior. Wilcoxon test showed this difference is significant (P<0.0001). Study of relationship between self care behavior and demographic data by Mannwitney U test showed in 15th day relationship between self care behavior and health assurance, job, history of hypertension and smoking is significant but in 30th day, correlation of self care behavior were significant with education status, health assurance, come back to job, hypertension history and other chronic diseases. Distribution and comparing of self care behavior in 15th and 30th day showed in Table 2.

**Table 2:** Self care in 15^th^ and 30^th^ day after MI

day Self care	Self care behavior in 30^th^ day		Self care behavior in 15^th^ day	
	
	N	%	N	%
indesire	111	84.1	37	28
desire	21	15.9	95	72
mean	72.50		84.46	
SD	11.70		9.62	
Test and significance	Wilcoxon test Z= - 8.35 P<0.0001			

Relationship between depression symptoms and self care behavior For study of this aim, Spearman test was done and showed this relationship in 15th and 30th day after MI was significant (P< 0.0001). Use of GEE model also showed in lack of depression symptoms as a reference group, in severity of depression symptoms, self care behavior would be decreased 2.63 times lower than patient without these symptoms (P<0.0001) (Table 3).

**Table 3:** Regression coefficients and odds ratio of depression symptoms and self care behavior relationship based on GEE

Parameter	Odd ratio		Relative chance	P value	SE	β

	< mean	mean<				
Severe depression symptoms confront to without depression symptom	3.12	2.22	2.63	0.001	0.087	0.968
Moderate depression symptoms confront to without depression symptom	2.51	1.61	2.02	0.001	0.1126	0.703
Mild depression symptoms confront to without depression symptom	1.34	1.32	1.33	0.001	0.0039	0.29
without depression symptom	Reference group					

This model also showed with control of demographic variables effects, self care behavior in severe depression symptoms 14.05 times lower than in patient without depression symptoms (Table 4).

**Table 4:** Regression coefficients and odds ratio of depression symptoms and self care behavior relationship based on GEE after demographic parameters control

Parameter	Odd ratio		Relative chance	P value	SE	β

	mean<	< mean				
Severe depression symptoms confront to without depression sympto	67.97	2.90	14.05	0.001	0.804	2.64
Moderate depression symptoms confront to without depression symptom	7.26	2.59	4.33	0.001	0.263	1.46
Mild depression symptoms confront to without depression symptom	3.11	1.44	2.11	0.001	0.196	0.751
without depression symptom	Reference group					

Furthermore finding of this model showed self care behavior in severe depression symptoms was 2.42 times lower than MI patient without depression symptoms (Table 5).

**Table 5:** Regression coefficients and odds ratio of depression symptoms and self care behavior relationship based on GEE after illness related parameters control

Parameter	Odd ratio		Relative chance	P value	SE	β

	< mean	mean<				
Severe depression symptoms confront to without depression symptom	2.55	2.29	2.42	0.0001	0.026	0.884
Moderate depression symptoms confront to without depression symptom	1.50	1.42	1.46	0.0001	0.014	0.381
Mild depression symptoms confront to without depression symptom	1.25	1.23	1.24	0.0001	0.004	0.222
without depression symptom	Reference group					

## 4. Discussion

### 4.1 Depression Symptoms After MI

In this study, depression symptoms in 30th day were increased in compare of these symptoms in 15th day after MI. It seems this finding may relate to increase patients′ perceive about their disease and it’s limitations and knowing that MI may go on to end of life. This finding is similar to Parashar etal findings that showed depression symptoms were increased in 30th day after MI in 31.1% of their participants (Parashar & Vaccarino, 2007). Spijkerman et al. (2006) also found depression symptoms was increased in 19th day after MI. It is noticeable that the tools were used for depression symptoms measuring in those studies were not similar to our tool.

In 15th day, mean of depression symptoms scores in male and female had statistical difference and women’s score was upper than men. Kronish et al. (2006) also found the majority of their participants who had depression symptoms were women. Finding of Chan et al. (2007) study also showed symptom of depression in women was more than men after MI. In Iran responsibility of women is much spread. They care of their children and grandchild and often work out of home. Many of middle age women work in farms, factories and offices. They manage all of their responsibilities about their home, children and work. It seems after MI, the women’s role in their home, and society would be decreased and this event can affect them and increasing depression symptoms. In our study was found mean scores of depression symptoms and having health insurance had statistical relationship. When patients don’t have insurance, have to pay all their care costs that always are so expensive, this situation may affect patients and may help to increase depression symptoms. Findings of Parashar et al. (2006) study also similar to our finding about insurance support. We found rural patient had depression symptom more than urban patients in 15th day after MI. Rural patients are far from medical centers and this issue can help to increase depression symptoms. This finding is similar to Modabberinia et al. (2001) findings about depression incidence after MI in north of Iran.

Using of statistical test about relationship between demographic variables and depression symptoms showed these symptoms were correlated only with history of other chronic diseases. This result may relate to this point, when patient have many illness, have to observance some care issue and may affect on his or her life style. This challenge can influence on depression symptoms.

### 4.2 Self Care Behaviors After MI

Our finding indicates after 15th and 30th day after MI, self care behavior had meaningful changes and by passing time score of self care behavior was decreased. It was similar to finding of Coyle et al. (2009) study who surveyed the changes of self care behavior in 15th and 30th day after MI. Passing days after MI may influence on patients perceive about their illness and they understand to have to do special behavior to help them. These changes may need more time and one month passing after MI was not enough for coping to new situation. In addition, results showed despite similarity of education about illness and it’s caring, patients with higher grade education had higher self care behaviors score. It is likeliness to finding of Heo et al. (2008). Thus of preparation and education base on educational level of each patient, can affect more and more on patient’s knowledge about self care and they can do these behaviors. Score in patients who have health insurance was better than others. Adherence of medical and drug regime is so expensive and if governments pay a section of its costs, help to do self care behavior in better manner. When patient know that his or her therapeutic and care costs is paid by insurance agency, adherence of medical regime will be simple. The findings of this study supports finding from Hiestand et al. (2004) about insurance status and treatment of myocardial infarction patients (Hiestand et al., 2004).

The findings indicated mean score of self care behavior was increased in MI patient with hypertension and smoking history before MI. These patients had to adhere of a special regime and after MI they familiar to self care behavior so they had mean score of self care more better than other patients. This finding underpins results of Ruo et al. (2006) about health among women with coronary disease.

No smoking is a domain of self care after MI and quitting smoking often is a difficult process for patients. So patients without smoking history can do better self care behavior after MI. Many of smokers say cigarette can reduce their stress and it is obvious with this opinion quitting smoking is difficult. This finding is reported by Gerber et al. (2009) study about smoking status and long term survival after first MI.

Domains of self care behavior consist of adherence to drug regimen, control of diet, exercise, performance, stress reduction and cessation of smoking. In our study expect drug regimen what did not have statistical meaningful difference in 15th and 30th day mean scores of other domains in 30th day were lower than in 15th day after MI. In Coyle (2009) study- who her study’s tool was used in our study – found mean score of cessation of smoking and drug regime were similar in 15th and 30th day after MI, but the mean score of other domains were reduced in 30th day compare to 15th day. After MI the energy and motivation of patients usually are decreased and they may need to more time to doing self care behavior, but 30 days may not be enough for these changes.

### 4.3 Depression Symptoms Effect on Self Care Behavior

In this study Spearman test showed statistical correlation between depression symptoms and self care behavior in 15th and 30th day after MI. This finding was different from Coyle etal finding about correlation of these variables in 15th day after MI, but was similar to their findings in 30th day after MI (Coyle, 2009). It is noticeable self care behavior is affected by culture and geographic area. In many of countries patients participate in a rehabilitation program after discharging from hospital that controls their behaviors. Unfortunately this setting is not in the city of this study was done in. It seems rehabilitation setting can help patients to commitment to self care behavior.

For predicting severity of depression symptoms effect on self care behavior, using of GEE model showed with control of all socio demographic and illness related factors, self care behavior in patients with severe depression symptoms is 14.05 times lower than other patients. This finding emphasis if all other factors control, depression symptoms can reduce to do self care behavior. This finding is similar the finding of Kronish et al.’s study. (2006).

### 4.4 Study Limitation

In this study measures were obtained from self reports and may reflect bias in reporting. Furthermore generalizing of our finding was limited, because our samples were chosen by convenience sampling that was none randomize sampling. Gathering data in 15th and 30th day after MI may form recall bias.

## 5. Conclusion

Data from this study are confirmed, despite of many of studies, beginning of depression symptoms is in 15th day after MI and increasing to 30th day. This important finding can use in nursing students educational programs to increase knowledge of nurses about mood changes of patients after MI. In addition finding of our study confirm necessity of rehabilitation program setting for these patients. This issue is important especially in cure and caring centers. Without rehabilitant setting, it seems use of community health and psychiatric nurse can useful.

The findings don’t generalize because samples of study were not chosen by randomized selection.
